# Ewing’s sarcoma of the cervix, a diagnostic dilemma: a case report and review of the literature

**DOI:** 10.1186/s13256-015-0733-2

**Published:** 2015-11-09

**Authors:** Nazia Mashriqi, Jaya kranthi Gujjarlapudi, Jagmohan Sidhu, Michael Zur, Madhuri Yalamanchili

**Affiliations:** 1 United Health Services Wilson Medical Center, Johnson, NY 13790 USA; 2 Broome Oncology, United Health Services Wilson Medical Center, Johnson, NY 13790 USA; 3 Our Lady of Lourdes Hospital, Binghamton, NY 13905 USA

**Keywords:** Ewing’s sarcoma, PNET, Uterine cervix

## Abstract

**Introduction:**

Ewing’s sarcoma belongs to a spectrum of neoplastic diseases known as Ewing’s family of tumors. This family of tumors is usually seen in osseous sites. Ewing’s sarcoma of the cervix is extremely rare, with only 18 cases reported in the English literature. The immunohistochemical profile of Ewing’s sarcoma overlaps with other malignancies like small cell carcinoma. The rarity and complex pathologic picture of Ewing’s sarcoma of the cervix creates the potential for misdiagnosis. Hence, we believe this case needs to be reported to add to the available literature.

**Case presentation:**

A 49-year-old white Caucasian woman presented with vaginal bleeding. A pelvic examination revealed a cystic lesion arising from her cervix. Examination of a biopsy specimen revealed a poorly differentiated neoplasm, with sheets of small hyperchromatic cells, staining weakly for neuroendocrine markers. She was diagnosed with small cell carcinoma and started on concurrent chemotherapy and radiation. However, additional positive immunostaining for CD99 was strongly suggestive of Ewing’s sarcoma. Fluorescence *in situ* hybridization revealed *ESWR1* gene rearrangement, confirming Ewing’s sarcoma. Our patient underwent surgery, which confirmed stage IIB Ewing’s sarcoma. She received adjuvant chemotherapy but died from progressive metastatic disease after four cycles.

**Conclusion:**

With early diagnosis and appropriate treatment, Ewing’s sarcoma of the cervix can be a potentially curable disease. However, owing to overlapping clinical and histopathological features, the diagnosis poses a challenge to oncologists and pathologists. This article guides pathologists to consider Ewing’s sarcoma in the differential diagnosis of small cell carcinoma with weak staining for neuroendocrine markers. This literature review will benefit oncologists encountering this rare entity.

## Introduction

Ewing’s sarcoma and peripheral neuroectodermal tumor (PNET) are the same entity, displaying varying degrees of neuroectodermal differentiation. They arise from mesenchymal progenitor cells and are part of a spectrum of neoplastic diseases, known as Ewing’s family of tumors (EFT) [1]. EFT are characterized by reciprocal translocation between chromosomes 11 and 22, t (11; 22), and are usually seen in osseous sites, both axial and appendicular. Extra-osseous presentations are uncommon, with specifically PNET of the female genital tract being very rare. The most common site of PNET in the female genital tract is the ovary, with the uterine corpus being the second most common. Primary PNET of the cervix and vulva are extremely rare. In this article, we present a case of primary PNET tumor of the cervix. The rarity of this entity can lead to diagnostic difficulties. In our case, it was initially diagnosed as a neuroendocrine small cell carcinoma of the cervix. We also summarize a literature review of all the cases reported in the English language.

## Case presentation

**Fig. 1:**
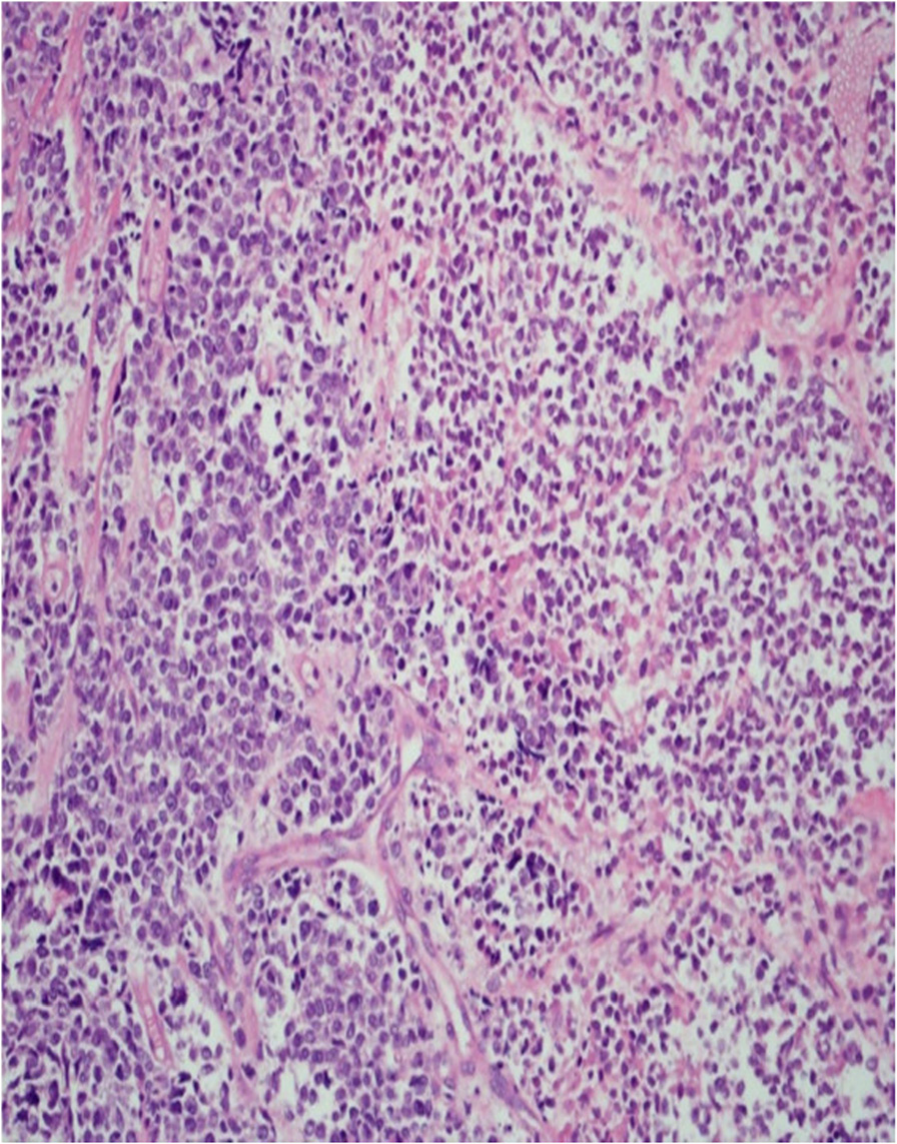
Medium power (200×) hematoxylin and eosin stain showing sheets of small tumor cells, focal necrosis, and a delicate vascular network

**Fig. 2:**
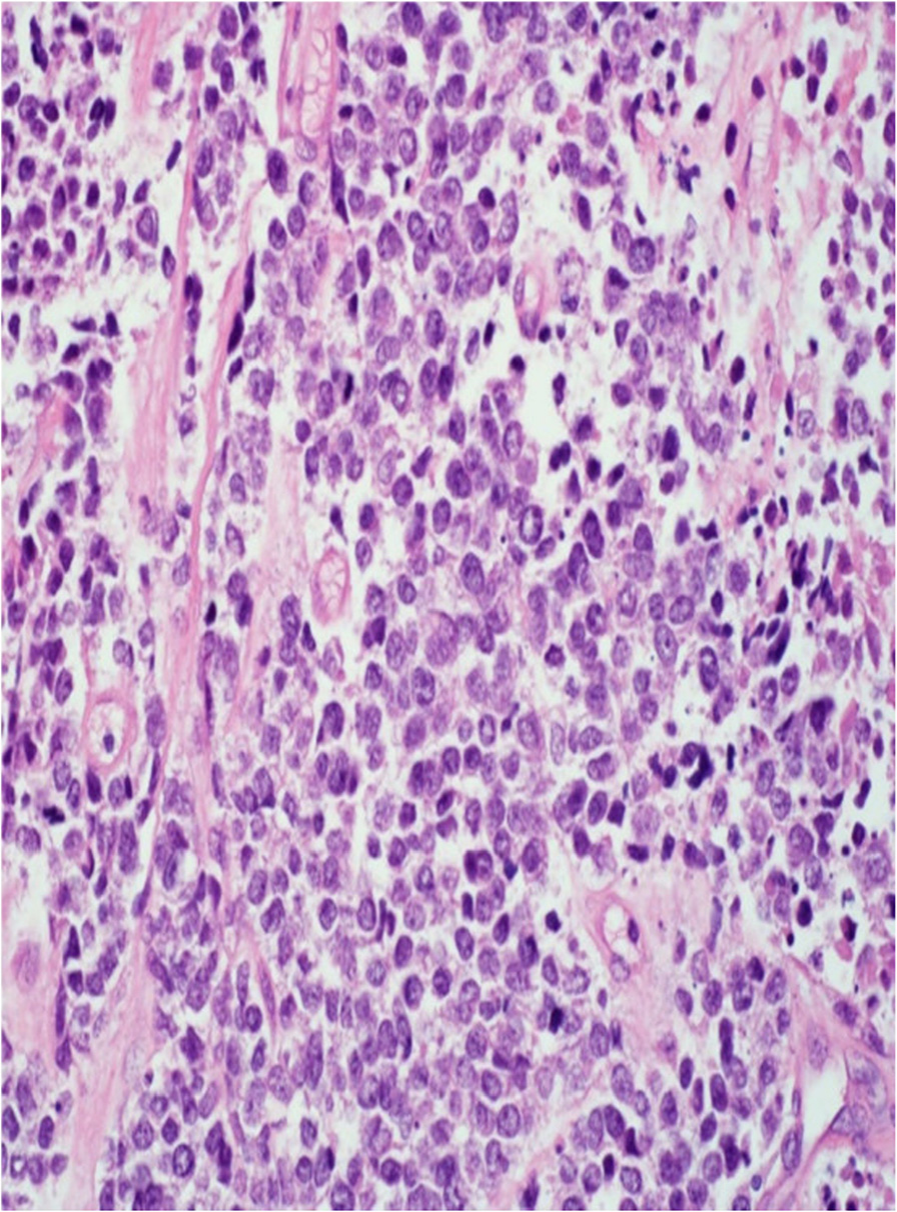
High power (400×) hematoxylin and eosin stain showing small cells with focal necrosis and mitotic activity

**Fig. 3:**
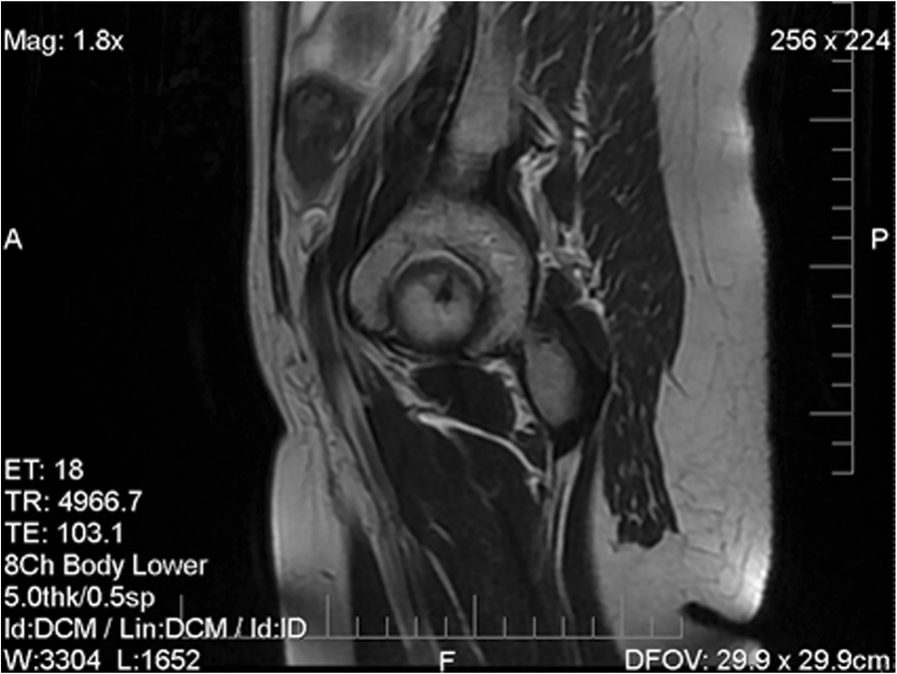
Magnetic resonance imaging of the pelvis: sagittal view. An enhancing mass is visible in the anterior cervix

A 49-year-old gravida 2, para 2, perimenopausal woman presented with vaginal bleeding. A pelvic examination revealed a cystic lesion arising from her cervix. Examination of a biopsy specimen revealed a poorly differentiated neoplasm involving the cervical stroma, with sheets of small hyperchromatic cells with slightly irregular nuclei, stippled chromatin, inconspicuous nucleoli, minimal cytoplasm, necrosis, and numerous mitotic figures (Figs. 1 and 2). Immunostaining showed the samples were weakly positive for neuron-specific enolase, CD56, and synaptophysin, and negative for pancytokeratin, CK7, CK20, and CD45. Magnetic resonance imaging (MRI) of the pelvis showed a 5.3 × 4.8 × 6.6 cm irregular, enhancing mass arising from her cervix, with involvement of the adjacent vagina and parametrium (Fig. 3). A positron emission tomography–computed tomography (PET/CT) scan revealed that the mass was hypermetabolic with a standard uptake value of 5.5, and did not demonstrate any distant metastatic disease.

**Fig. 4:**
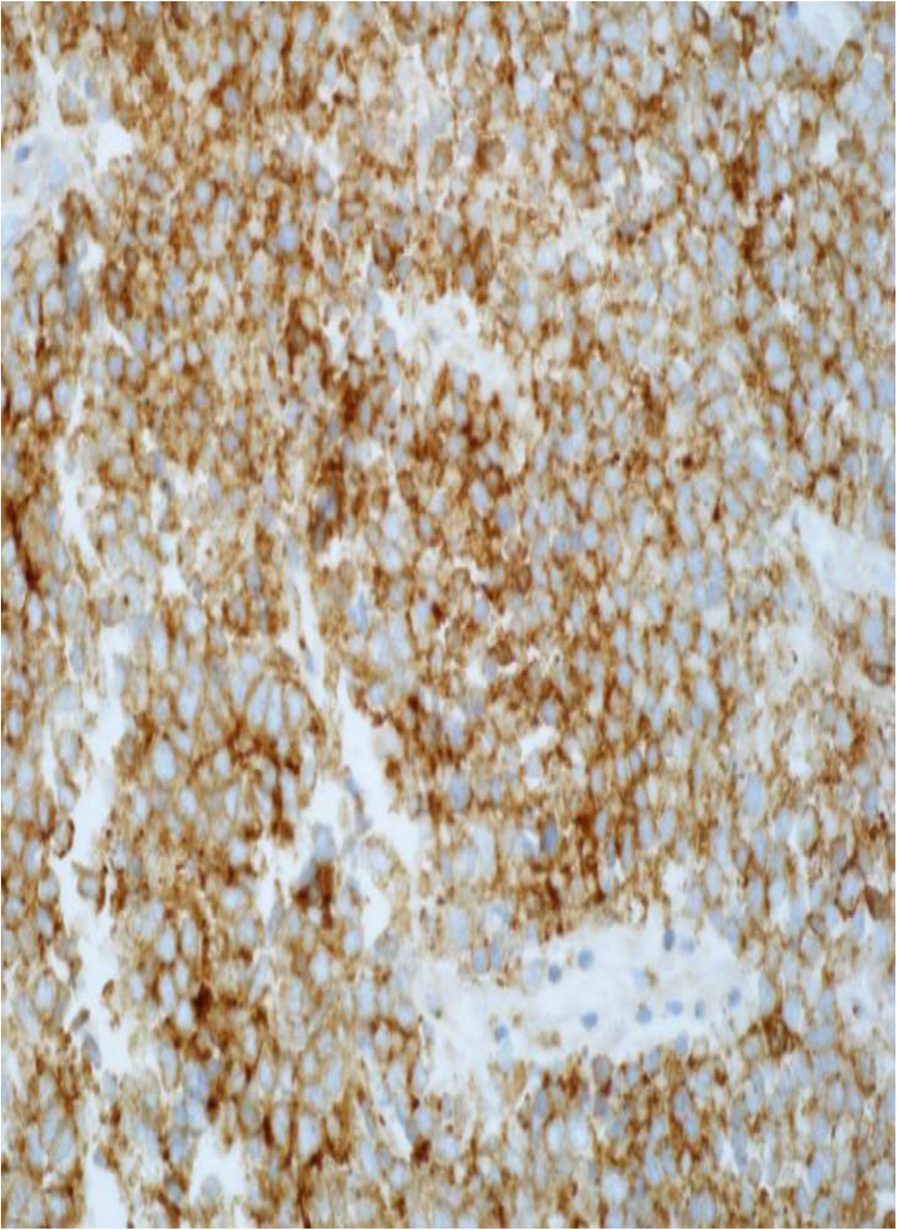
High power (400×) immunohistochemical diffuse and membranous CD99 positivity

The pathologic diagnosis was invasive malignant small round blue cell tumor most consistent with small cell carcinoma. She completed two cycles of cisplatin and etoposide and a planned course of 5,040 cGy over 28 fractions of 180 cGy each, which led to resolution of the vaginal bleeding. However, owing to the only weak staining for the neuroendocrine markers, further immunostaining was performed. Stains for S-100, CD3, CD20, TTF1, and desmin were negative whereas that for CD99 was strongly positive (Fig. 4). Diffuse membranous positivity for CD99 led to a diagnosis of Ewing’s sarcoma/PNET and ruled out small cell carcinoma. Fluorescence *in situ* hybridization revealed *ESWR1* gene rearrangement in 90 % of cells, confirming the diagnosis of Ewing’s sarcoma. Subsequently, chemotherapy was discontinued and our patient underwent total hysterectomy and bilateral salpingo-oophorectomy, upper vaginectomy, and resection of the parametrium. Surgical pathology showed stage IIB Ewing’s sarcoma involving her cervix and extending into the right parametrium. Her endometrium, ovaries, and fallopian tubes were unremarkable and margins were free of tumor.

She was started on chemotherapy with vincristine (2 mg/m^2^ on day 1), doxorubicin (Adriamycin; 75 mg/m^2^), and cyclophosphamide with mesna (1,200 mg/m2 on day 1), alternating with ifosfamide plus mesna (1,800 mg/m^2^ days 1–5) and etoposide (100 mg/m^2^) every 3 weeks. A PET scan done after two cycles demonstrated no recurrence of the tumor. After four cycles, 9 months from initial diagnosis, she developed acute renal failure. CT scans showed evidence of metastases to her lumbar spine, pelvis, and bladder. Nephrostomy tubes were placed, which improved renal function. Two weeks later she presented with distal colonic obstruction secondary to an extensive pelvic tumor. Exploratory laparotomy and a diverting loop colostomy were done. One week later her disease progressed with new lung metastases, which led to clinical deterioration and death, with an overall survival of 10 months.

## Discussion

Ewing’s sarcoma/PNET of the cervix is an extremely rare entity. Our review shows that there have been only 18 cases reported so far in the English literature; we report the 19th case (Table 1). In two cases, PNET was reported in association with another malignancy. Horn *et al*. reported PNET with squamous cell carcinoma of the cervix, and Tsao *et al*. reported carcinoma in addition to PNET, and described their case as a “carcinosarcoma” [2, 3]. The ages at presentation ranged between 19 and 60 years, with the mean age being 39.

**Table 1:** Clinical and pathologic features, diagnosis, treatment, and outcome of peripheral neuroectodermal tumors of the cervix

	Author	Age	Symptoms	Diagnosis	Stage	Surgery	Chemotherapy/intent of chemotherapy	Radiation therapy	Outcome
1	Russin *et al*. 1987 [21]	60	Vaginal bleeding	Path/IHC	IB	TAH + BSO + LND	VAC for 6 weeks/adjuvant	Yes	Alive at 16 months, NED
2	Sato *et al*. 1996 [22]	44	Irregular vaginal bleeding	Path/IHC	IB2	TAH + BSO + LND, second look after 6 months	Cisplatin, VP16, cyclophosphamide (Cytoxan), doxorubicin (Adriamycin)/adjuvant	No	Alive 6 months, NED
3	Horn *et al*. 1997 [2]	26	Suspect cervical smear	Path/IHC	IB1	TAH + BSO + LND	No adjuvant chemotherapy; had lung metastases 3 years after diagnosis, received 5FU and cisplatin/palliative	RT to metastases	Died 4.2 years after diagnosis
4	Cenacchi *et al*. 1998 [15]	36	Irregular vaginal bleeding	Path/IHC/RT-PCR	IB2	TAH without BSO	No	No	Alive 18 months, NED
5	Pauwels *et al*. 2000 [14]	45	Irregular vaginal bleeding	Path/IHC/FISH	IB2	TAH	No	Pelvic RT	Alive 42 months, NED
6	Tsao *et al*. 2001 [3]	24	Vaginal bleeding, urinary frequency	Path/IHC		TAH + transposition of ovaries + LNS	Two cycles, VAC alternating with IE, neoadjuvant and adjuvant	Yes	Alive 24 moths, NED
7	Malpica and Moran 2002 [6]	35	Vaginal bleeding	Path/IHC	IB1	TAH + BSO + LND	Adjuvant chemotherapy/regimen not reported	No	Alive 5 months, NED
8	Malpica and Moran 2002 [6]	51	Vaginal bleeding	Path/IHC	IB2	TAH + BSO + LND	Adjuvant chemotherapy/regimen not reported	No	Alive 18 months, NED
9	Snijders-Keilholz and Ewing 2005 [17]	21	Intermenstrual bleeding	Path/IHC	IB2	TAH without adnexectomy	Six courses of DIME/neoadjuvant; five courses of VIA/adjuvant	No	Alive 27 months, NED
10	Goda *et al*. 2007 [19]	19	Vaginal bleeding, discharge			No	Induction VAC, planned for further consolidation after RT	Yes	Alive, on treatment when reported
11	Farzaneh *et al*. 2011 [23]	43	Purulent vaginal discharge	Path/IHC	IB2	TAH + BSO + LNS	12 weeks of VAC alternating with IE/neoadjuvant	No	Alive 4 years, NED
							12 weeks of VAC alternating with IE/adjuvant		
12	Benbrahim *et al*. 2012 [9]	25	Irregular vaginal bleeding	Path/IHC	IIb	Coniztion with brachytherapy	Four cycles of Adriamycin and Cytoxan/neoadjuvant	Yes	Alive 8 years, NED
13	Arora *et al*. 2012 [4]	23	Irregular bleeding, dysuria	Path/IHC		TAH + BSO + LND	One cycle of CAV, followed by two cycles of cis/VP16/neoadjuvant	Yes	Alive 4 years, NED
14	Masoura *et al*. 2012 [16]	23	Irregular bleeding, abdominal pain	Path/IHC/RT-PCR	IV	TAH + BSO	Cisplatin once/adjuvant	No.	Died, 12 days
15	Li *et al*. 2013 [5]	27	Contact bleeding, abdominal pain	Path/IHC	IIIB	Unresectable	VAC alternating with IE/definitive chemotherapy	Yes	Alive at 6 months, NED
16	Khosla *et al*. 2014 [24]	28	10 weeks pregnant with vaginal bleeding and pelvic pain	Path/IHC	IB2	Termination of pregnancy, TAH + BSO + LNS	Adriamycin, IE, for total of 6 weeks/adjuvant	No	Alive 33 months, NED
17	Xiao *et al*. 2014 [18]	52	Vaginal bleeding, uterine enlargement	Path/IHC	IIA	TAH + BSO + LND	Two courses of PVB		Pelvic recurrence 6 months, DOD 9 months
18	Xiao *et al*. 2014 [18]	59	Cervix prolapse, vaginal bleeding	Path/IHC	IVB	TAH + BSO + LND	None		DOD
19	Present case	49	Vaginal bleeding, lower abdominal pain	Path/IHC/FISH	IIB	TAH + BSO	Cisplatin/Etoposide with RT due to diagnosis of small cell VAC alternating with IE/adjuvant	Yes	Died, 10 months

*5FU* 5-fluorouracil, *BSO* bilateral salpingo oophorectomy, *DIME* Doxorubicin, Ifosfamide, Mesna, Etoposide, *DOD* died of disease, *FISH* fluorescent *in situ* hybridization, *IE* Ifosfamide, Etoposide, *IHC* immunohistochemical studies, *LND/LNS* pelvic lymphadenectomy/lymph node sampling, *LSO* left-sided oophorectomy, *NED* no evidence of disease, *PVB* Cisplatin, Vincristine, Bleomycin, *RT* radiation therapy, *RT-PCR* reverse transcriptase polymerase chain reaction, *TAH* total abdominal hysterectomy, *VAC* Vincristine, Adriamycin, Cyclophosphamide, *VIA* Vincristine, Ifosfamide, Dactinomycin, *VP16* Etoposide

The most common symptom reported was irregular vaginal bleeding. Other symptoms included dysuria, lower abdominal pain, vaginal discharge, and in one case, urinary frequency. The most common physical findings were nodular lesions extending into the anterior vaginal wall and enlarged uterus. The vaginal bleeding with enlarged uterus led to the preliminary diagnosis of fibroid in two patients [3, 4]. One patient had vaginal stenosis and necrotic tissue on the cervix [5].

Stage was not reported in three cases. Ten (62.5 %) patients were stage IB1 or IB2, one (6.25 %) stage IIA, two (12.5 %) stage IIB, one (6.25 %) stage IIIB, and two stage IV (12.5 %).

In the reported cases, multiple imaging modalities including ultrasound, CT, and MRI were used for diagnosis and staging. Our review shows that PNET tumors are highly fludeoxyglucose avid. PET scan may be a useful imaging modality in diagnosis, staging, and monitoring response to therapy.

The diagnosis of EFTs is difficult by routine microscopy because they have small blue cell morphology that can be seen in several malignancies. On histology examination, there are sheets of small blue cells with “stippled salt and pepper chromatin” in the nuclei and absence of nucleoli [6]. Necrosis and nuclear molding of adjacent cells is common. Additionally, the cells easily become crushed during processing of the specimen, producing smudged and streaked extra nuclear chromatin (crush artifact). Small round blue cells can be seen in a wide variety of malignancies which can be remembered using the pneumonic “LEMON” (lymphoblastic lymphoma, Ewing’s sarcoma, medulloblastoma, oat cell/small cell neuroendocrine, and neuroblastoma). Other soft tissue sarcomas and rhabdomyosarcoma should also be considered in the differential diagnosis. Lymphoblastic lymphomas closely resemble PNET because they have sheets of small cells with a lack of glandular or squamous differentiation.

Immunohistochemistry is critical in the diagnosis. Small cell neuroendocrine carcinomas are usually positive for chromogranin, synaptophysin, and neuron-specific enolase, and overlap highly with PNET. The vast majority of EFTs express high levels of a cell surface glycoprotein CD99 or MIC2 surface antigen that is encoded by the CD99 (MIC2X) gene [7, 8]. The finding of membrane-localized MIC2 expression in a small blue cell malignancy is a sensitive diagnostic marker for the EFTs. MIC2 lacks specificity because other tumors, like rhabdomyosarcoma, can be MIC2 positive. However, small cell carcinomas are negative for MIC2. Benbrahim *et al*. reported a case of cervical PNET that was initially misdiagnosed as lymphoma [9]. Lymphoid markers, leucocyte common antigen, CD20, and CD3 can be utilized to differentiate lymphoma from PNET [9].

Molecular genetic characterization of chromosomal anomalies specific to EFT has led to increased detection. The characteristic signature translocation involving the *EWS* gene at 22q12.2 and various erythroblast transcription specific-family genes, like *FLI* (friend leukemia virus integration 1) at 11q24.1-q24.3, is seen in 85–90 % of cases [10, 11]. *ESWR* encodes a multifunctional protein that regulates multiple cellular processes. *FLI1* encodes the FLI1 protein, which controls cellular development, proliferation, and carcinogenesis [12]. The *EWSR1*–*ERG* translocation [t (21; 22) (q22; q12)] is present in 5–10 % of EFTs, while other translocations are less common [13].

Most of the cases reported were diagnosed based on histopathology and immunohistochemistry (Table 1). Cytogenetic analysis [14] was done in one case. Fluorescent *in situ* hybridization [14] was used in two cases including our case, while reverse transcriptase polymerase chain reaction [15, 16] was used in two cases.

Currently there is no uniformity of treatment, owing to the rarity of this neoplasm. Snijders-Keilholz *et al*. recommended a multidisciplinary approach similar to that used in osseous PNETs with induction chemotherapy, surgery, adjuvant chemotherapy, and radiation [17]. When surgery is feasible, wide excision performed at a sarcoma center is preferable.

Most patients (Table 1) with early stage disease underwent total abdominal hysterectomy and bilateral salpingo-oophorectomy. Pelvic lymph node dissection was performed in 11 out of 19 cases. Of these 11 cases, eight patients are alive without recurrence, the outcome of two cases is unknown, and one patient died from metastatic disease 4 years later. Of the eight cases that did not use lymph node dissection, two tumors were inoperable owing to stage IIIB [5] and IV [16] disease. The stage of one patient is unknown. The remaining five cases were early stage (three stage I B2, two stage IIB). Of these five cases, one patient did not receive any chemotherapy and died 4 years later. Our patient (stage IIB) died 10 months later despite chemotherapy and radiation. The other three cases received chemotherapy and are alive without relapse. The contribution of Pelvic lymph node dissection (PLND) to overall survival, especially in patients who had chemoradiation, is unclear.

Chemotherapy was used in 16 of the 19 cases (Table 1). Two patients who were metastatic at diagnosis received palliative chemotherapy. Although adjuvant chemotherapy was used in the earlier reported cases, a combined regimen of neoadjuvant and adjuvant chemotherapy has been used frequently in recent years, with favorable results. The chemotherapy regimens and schedules used varied considerably. The use of ifosfamide and etoposide alternating with vincristine, doxorubicin (Adriamycin) and cyclophosphamide, which is the regimen of choice for Ewing’s sarcoma of the bone, has increased in recent years with good outcomes. The overall survival appears to have dramatically improved with the use of chemotherapy.

Eight of 19 patients (Table 1) received radiation therapy. The intent of radiation was palliative in one case, definitive in three, and adjuvant in four. The definitive chemoradiation given to our patient was part of the small cell carcinoma regimen owing to an initial small cell diagnosis, but she subsequently developed pelvic recurrence 9 months after diagnosis. Another case death from pelvic recurrence 9 months after diagnosis was reported by Xiao *et al*. [18], but the authors did not report if radiation was given. Radiation doses ranged from 40 to 55 Gy with the fractionation schedule of 180–200 cGy over 4–5 weeks. Overall, we conclude that adjuvant radiation may have a role in preventing local recurrence and should be considered when appropriate.

The follow-up of these cases ranged between 5 months and 8 years, with 15 of the 19 cases being alive and recurrence-free at the time of follow-up. Two patients died of metastatic disease 12 days and 4.2 years after presentation, respectively. Our patient developed metastatic disease while on adjuvant chemotherapy and died 10 months after diagnosis. The outcome of a case reported by Goda *et al*. [19] is unknown.

The best outcomes were noted in patients who underwent tri-modality therapy with surgery, chemotherapy, and radiation; although owing to the paucity of cases the best approach is still unknown.

Similar to skeletal Ewing’s sarcoma, the most unfavorable prognostic factor is the presence of distant metastasis, with stage IV disease being universally fatal. A recent review by Baldini *et al*. [20] suggested that age may be a prognostic factor in survival and elderly patients do poorly.

## Conclusion

It is important to identify these rare cases of Ewing’s sarcoma to provide early and appropriate treatment. With prompt diagnosis and aggressive multimodality treatment, PNET of the cervix appears to be a potentially curable disease. Once diagnosis is made, referral to a tertiary care center with dedicated multidisciplinary tumor boards and special expertise in sarcoma management is recommended. Hopefully, identification of more cases in the future may help establish a meaningful pattern of behavior and guide clinical management of this rare entity.

## Consent

Written informed consent was obtained from the patient’s next of kin for publication of this case report and any accompanying images. A copy of the written consent is available for review by the Editor-in-Chief of this journal.
